# 3,6-Bis(3,4,5-trimethoxy­phen­yl)-1,2,4-triazolo[3,4-*b*][1,3,4]thia­diazole

**DOI:** 10.1107/S1600536808022502

**Published:** 2008-07-23

**Authors:** Hai-Tang Du, Hai-Jun Du, Weiyi Zhou

**Affiliations:** a Institute of Natural Products, Research Center for Eco-Environmental Sciences, Guiyang College, Guiyang 550005, People’s Republic of China; b School of Chemistry and Environmental Sciences, Guizhou University for Nationalities, Guiyang 550025, People’s Republic of China; c Analytical Center, Tianjin University, Tianjin 300072, People’s Republic of China

## Abstract

In the mol­ecule of the title compound, C_21_H_22_N_4_O_6_S, the planar central heterocyclic ring system is oriented with respect to the trimethoxy­phenyl rings at dihedral angles of 2.60 (5) and 3.60 (6)°. Intra­molecular C—H⋯N and C—H⋯S hydrogen bonds result in the formation of planar five- and six-membered rings. In the crystal structure, inter­molecular C—H⋯O hydrogen bonds link the mol­ecules. There is a C—H⋯π contact between a methyl group and a trimethoxy­phenyl ring, and a π–π contact between the central heterocyclic ring system and a trimethoxy­phenyl ring [centroid–centroid distance = 3.640 (1) Å].

## Related literature

For general background, see: Karabasanagouda *et al.* (2007 ▶); Mathew *et al.* (2007 ▶).
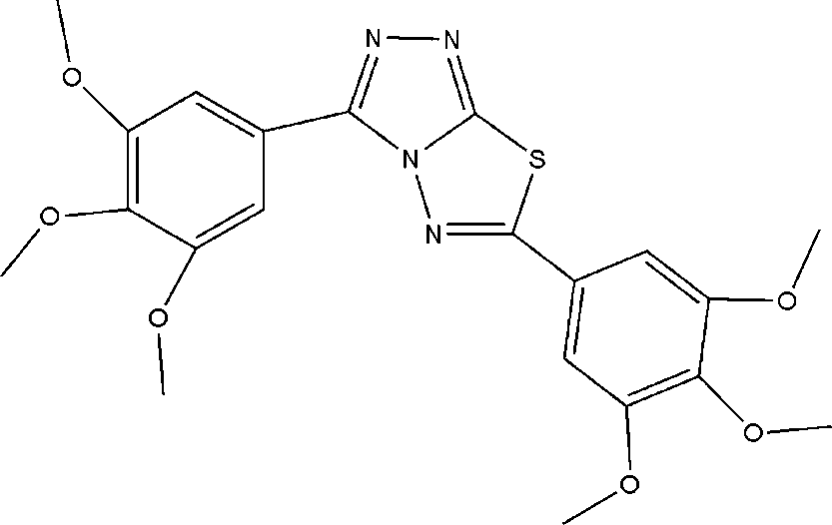


## Experimental

### 

#### Crystal data

C_21_H_22_N_4_O_6_S
*M* *_r_* = 458.49
Triclinic, 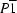

*a* = 8.6762 (17) Å
*b* = 8.9289 (18) Å
*c* = 13.735 (3) Å
α = 94.50 (3)°
β = 90.82 (3)°
γ = 90.47 (3)°
*V* = 1060.6 (4) Å^3^
*Z* = 2
Mo *K*α radiation
μ = 0.20 mm^−1^
*T* = 113 (2) K
0.22 × 0.20 × 0.10 mm

#### Data collection

Rigaku Saturn CCD area-detector diffractometer
Absorption correction: multi-scan (*CrystalClear*; Rigaku/MSC, 2005 ▶) *T*_min_ = 0.957, *T*_max_ = 0.980
6899 measured reflections
3720 independent reflections
3102 reflections with *I* > 2σ(*I*)
*R*_int_ = 0.022

#### Refinement

*R*[*F* ^2^ > 2σ(*F* ^2^)] = 0.044
*wR*(*F* ^2^) = 0.137
*S* = 1.19
3720 reflections
295 parameters
H-atom parameters constrained
Δρ_max_ = 0.93 e Å^−3^
Δρ_min_ = −0.56 e Å^−3^

### 

Data collection: *CrystalClear* (Rigaku/MSC, 2005 ▶); cell refinement: *CrystalClear*; data reduction: *CrystalStructure* (Rigaku/MSC, 2005 ▶); program(s) used to solve structure: *SHELXS97* (Sheldrick, 2008 ▶); program(s) used to refine structure: *SHELXL97* (Sheldrick, 2008 ▶); molecular graphics: *SHELXTL* (Sheldrick, 2008 ▶); software used to prepare material for publication: *SHELXTL*.

## Supplementary Material

Crystal structure: contains datablocks I, global. DOI: 10.1107/S1600536808022502/hk2498sup1.cif

Structure factors: contains datablocks I. DOI: 10.1107/S1600536808022502/hk2498Isup2.hkl

Additional supplementary materials:  crystallographic information; 3D view; checkCIF report

## Figures and Tables

**Table 1:** Hydrogen-bond geometry (Å, °)

*D*—H⋯*A*	*D*—H	H⋯*A*	*D*⋯*A*	*D*—H⋯*A*
C2—H2⋯N4	0.93	2.36	3.047 (3)	130
C14—H14⋯S1	0.93	2.72	3.130 (3)	108
C19—H19*B*⋯O3^i^	0.96	2.53	3.384 (2)	148
C21—H21*B*⋯O1^ii^	0.96	2.43	3.331 (3)	156
C19—H19*C*⋯*Cg*3^iii^	0.96	3.30	4.057 (3)	137

Symmetry codes: (i) 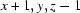; (ii) 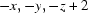; (iii) 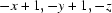. *Cg*3 is the centroid of the trimethoxyphenyl ring C1–C6.

**Fig. 1.**
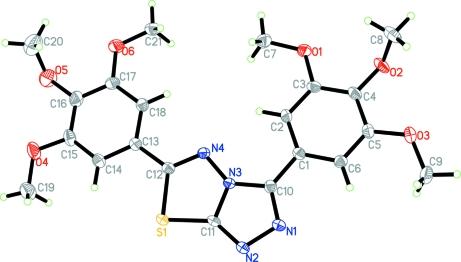
The molecular structure of the title molecule, with the atom-numbering scheme. Displacement ellipsoids are drawn at the 50% probability level.

**Fig. 2.**
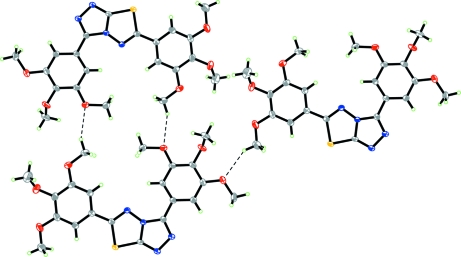
A partial packing diagram of the title compound. Hydrogen bonds are shown as dashed lines.

## supplementary crystallographic information

### Comment

1,2,4-Triazole and 1,3,4-thiadiazole represent one of the most biologically active classes of compounds, possessing a wide spectrum of activities. Various substituted 1,2,4-triazolo[3,4-b]-1,3,4-thiadiazoles are associated with diverse pharmacological activities such as antimicrobial (Karabasanagouda *et al.*,  2007) and anti-inflammatory activity (Mathew *et al.*,  2007). We report herein the crystal structure of the title compound.

In the molecule of the title compound (Fig. 1) the bond lengths and angles are within normal ranges. Rings A (C1-C6), B (N1-N3/C10/C11), C (S1/N3/N4/C11/C12) and D (C13-C18) are, of course, planar, and the dihedral angles between them are A/B = 3.42 (6)°, A/C = 1.96 (5)°, A/D = 4.76 (5)°, B/C = 1.65 (6)°, B/D = 3.91 (6)° and C/D = 3.42 (5)°. So, the rings are nearly coplanar. The intramolecular C-H···N and C-H···S hydrogen bonds (Table 1) result in the formation of planar six- and five-membered rings E: (N3/N4/C1/C2/C10/H2) and F (S1/C12-C14/H14), in which they are oriented with respect to the planar central heterocylic ring system at dihedral angles of 1.56 (5)° and 4.00 (5)°, respectively.

In the crystal structure, intermolecular C-H···O hydrogen bonds (Table 1) link the molecules (Fig. 2), in which they may be effective in the stabilization of the structure. A C—H···π contact (Table 1) between the trimethoxyphenyl ring and the methyl group and a π—π contact between B and D rings Cg2···Cg4^i^ [symmetry code: (i) 1 - x, 1 - y, -z, where Cg2 and Cg4 are centroids of the rings B (N1-N3/C10/C11) and D (C13-C18), respectively] further stabilize the structure, with centroid-centroid distance of 3.640 (1) Å.

### Experimental

For the preparation of the title compound, 4-amino-5-(3,4,5-trimethoxyphenyl) -4H-1,2,4-triazole-3-thiol (0.01 M) and 3,4,5-trimethoxybenzoic acid (0.01 M) were dissolved in dry phosphorous oxychloride (10 ml). The resulted solution was further heated under reflux for 7 h. The reaction mixture was cooled to room temperature and the mixture was gradually poured onto crushed ice with stirring. Finally, powdered potassium carbonate and the required amount of solid potassium hydroxide were added until the pH of the mixture was raised to 8, to remove the excess of phosphorous oxychloride. The mixture was allowed to stand overnight and the solid was separated. It was filtered, washed with cold water, and then dried. Crystals suitable for X-ray analysis were obtained by the recrystallization of the solid residue from a mixture of N,N-dimethyl- formamide/ethanol (1:1) by slow evaporation at room temperature.

### Refinement

H atoms were positioned geometrically, with C-H = 0.93 and 0.96 Å for aromatic and methyl H, respectively, and constrained to ride on their parent atoms with U_iso_(H) = xU_eq_(C), where x = 1.5 for methyl H and x = 1.2 for aromatic H atoms.

### Crystal data

**Table:**

C_21_H_22_N_4_O_6_S	*Z* = 2
*M**_r_* = 458.49	*F*_000_ = 480
Triclinic, *P*1	*D*_x_ = 1.436 Mg m^−^^3^
Hall symbol: -P 1	Melting point: 423 K
*a* = 8.6762 (17) Å	Mo *K*α radiation λ = 0.71073 Å
*b* = 8.9289 (18) Å	Cell parameters from 3022 reflections
*c* = 13.735 (3) Å	θ = 2.8–27.9º
α = 94.50 (3)º	µ = 0.20 mm^−^^1^
β = 90.82 (3)º	*T* = 113 (2) K
γ = 90.47 (3)º	Prism, colorless
*V* = 1060.6 (4) Å^3^	0.22 × 0.20 × 0.10 mm

### Data collection

**Table:**

Rigaku Saturn CCD area-detector diffractometer	3720 independent reflections
Radiation source: rotating anode	3102 reflections with *I* > 2σ(*I*)
Monochromator: confocal	*R*_int_ = 0.022
*T* = 113(2) K	θ_max_ = 25.0º
ω scans	θ_min_ = 2.8º
Absorption correction: multi-scan(CrystalClear; Rigaku/MSC, 2005)	*h* = −7→10
*T*_min_ = 0.957, *T*_max_ = 0.980	*k* = −10→10
6899 measured reflections	*l* = −15→16

### Refinement

**Table:**

Refinement on *F*^2^	Secondary atom site location: difference Fourier map
Least-squares matrix: full	Hydrogen site location: inferred from neighbouring sites
*R*[*F*^2^ > 2σ(*F*^2^)] = 0.044	H-atom parameters constrained
*wR*(*F*^2^) = 0.137	*w* = 1/[σ^2^(*F*_o_^2^) + (0.0797*P*)^2^ + 0.2575*P*] where *P* = (*F*_o_^2^ + 2*F*_c_^2^)/3
*S* = 1.19	(Δ/σ)_max_ = 0.003
3720 reflections	Δρ_max_ = 0.93 e Å^−^^3^
295 parameters	Δρ_min_ = −0.55 e Å^−^^3^
Primary atom site location: structure-invariant direct methods	Extinction correction: none

### Special details

**Table:**

Geometry. All e.s.d.'s (except the e.s.d. in the dihedral angle between two l.s. planes) are estimated using the full covariance matrix. The cell e.s.d.'s are taken into account individually in the estimation of e.s.d.'s in distances, angles and torsion angles; correlations between e.s.d.'s in cell parameters are only used when they are defined by crystal symmetry. An approximate (isotropic) treatment of cell e.s.d.'s is used for estimating e.s.d.'s involving l.s. planes.
Refinement. Refinement of F^2^ against ALL reflections. The weighted R-factor wR and goodness of fit S are based on F^2^, conventional R-factors R are based on F, with F set to zero for negative F^2^. The threshold expression of F^2^ > 2sigma(F^2^) is used only for calculating R-factors(gt) etc. and is not relevant to the choice of reflections for refinement. R-factors based on F^2^ are statistically about twice as large as those based on F, and R- factors based on ALL data will be even larger.

### Fractional atomic coordinates and isotropic or equivalent isotropic displacement parameters (Å^2^)

**Table:**

	*x*	*y*	*z*	*U*_iso_*/*U*_eq_	
S1	0.50010 (6)	0.76590 (6)	0.89162 (4)	0.01987 (19)	
O1	−0.04182 (18)	0.27249 (17)	1.14986 (12)	0.0270 (4)	
O2	−0.17317 (18)	0.40003 (18)	1.31069 (12)	0.0282 (4)	
O3	−0.09095 (19)	0.67584 (18)	1.38496 (11)	0.0290 (4)	
O4	0.6387 (2)	0.3996 (2)	0.57836 (12)	0.0352 (4)	
O5	0.5139 (3)	0.1310 (2)	0.59409 (12)	0.0457 (5)	
O6	0.3352 (2)	0.07371 (18)	0.74567 (12)	0.0305 (4)	
N1	0.2808 (2)	0.88200 (19)	1.13007 (13)	0.0207 (4)	
N2	0.3818 (2)	0.9307 (2)	1.06090 (13)	0.0220 (4)	
N3	0.31915 (19)	0.69318 (18)	1.02331 (12)	0.0161 (4)	
N4	0.3240 (2)	0.56265 (19)	0.96337 (12)	0.0173 (4)	
C1	0.1406 (2)	0.6468 (2)	1.16140 (15)	0.0175 (4)	
C2	0.1044 (2)	0.5002 (2)	1.12685 (15)	0.0197 (5)	
H2	0.1477	0.4583	1.0695	0.024*	
C3	0.0031 (2)	0.4170 (2)	1.17875 (16)	0.0204 (5)	
C4	−0.0631 (2)	0.4788 (2)	1.26440 (16)	0.0209 (5)	
C5	−0.0226 (2)	0.6260 (2)	1.29933 (15)	0.0205 (5)	
C6	0.0775 (2)	0.7102 (2)	1.24824 (14)	0.0193 (5)	
H6	0.1029	0.8082	1.2713	0.023*	
C7	0.0400 (3)	0.1984 (3)	1.07097 (18)	0.0311 (6)	
H7A	0.0241	0.2505	1.0131	0.047*	
H7B	0.0029	0.0969	1.0596	0.047*	
H7C	0.1480	0.1981	1.0871	0.047*	
C8	−0.1128 (3)	0.3006 (3)	1.37736 (19)	0.0356 (6)	
H8A	−0.0456	0.2300	1.3435	0.053*	
H8B	−0.1961	0.2475	1.4051	0.053*	
H8C	−0.0561	0.3572	1.4285	0.053*	
C9	−0.0618 (3)	0.8283 (3)	1.41985 (17)	0.0354 (6)	
H9A	0.0469	0.8435	1.4308	0.053*	
H9B	−0.1152	0.8504	1.4800	0.053*	
H9C	−0.0976	0.8936	1.3722	0.053*	
C10	0.2438 (2)	0.7397 (2)	1.10791 (15)	0.0184 (5)	
C11	0.4024 (2)	0.8140 (2)	0.99828 (15)	0.0178 (4)	
C12	0.4137 (2)	0.5860 (2)	0.89114 (15)	0.0175 (4)	
C13	0.4421 (2)	0.4679 (2)	0.81339 (15)	0.0179 (5)	
C14	0.5310 (2)	0.4968 (3)	0.73302 (15)	0.0218 (5)	
H14	0.5742	0.5916	0.7283	0.026*	
C15	0.5545 (3)	0.3832 (3)	0.66039 (16)	0.0260 (5)	
C16	0.4910 (3)	0.2408 (3)	0.66800 (16)	0.0285 (5)	
C17	0.3990 (3)	0.2149 (2)	0.74754 (16)	0.0233 (5)	
C18	0.3746 (2)	0.3271 (2)	0.82064 (15)	0.0201 (5)	
H18	0.3140	0.3090	0.8739	0.024*	
C19	0.6870 (3)	0.5477 (3)	0.56178 (19)	0.0390 (6)	
H19A	0.5995	0.6131	0.5647	0.058*	
H19B	0.7325	0.5476	0.4985	0.058*	
H19C	0.7615	0.5827	0.6109	0.058*	
C20	0.5942 (4)	−0.0017 (3)	0.6225 (2)	0.0512 (8)	
H20A	0.6491	0.0223	0.6830	0.077*	
H20B	0.6657	−0.0343	0.5727	0.077*	
H20C	0.5207	−0.0806	0.6307	0.077*	
C21	0.2401 (3)	0.0431 (3)	0.82573 (17)	0.0280 (5)	
H21A	0.2991	0.0570	0.8855	0.042*	
H21B	0.2029	−0.0587	0.8171	0.042*	
H21C	0.1543	0.1105	0.8286	0.042*	

### Atomic displacement parameters (Å^2^)

**Table:**

	*U*^11^	*U*^22^	*U*^33^	*U*^12^	*U*^13^	*U*^23^
S1	0.0218 (3)	0.0164 (3)	0.0217 (3)	−0.0034 (2)	0.0056 (2)	0.0029 (2)
O1	0.0264 (9)	0.0190 (8)	0.0353 (9)	−0.0065 (7)	0.0089 (7)	−0.0005 (7)
O2	0.0202 (8)	0.0324 (9)	0.0340 (9)	−0.0024 (7)	0.0086 (7)	0.0152 (7)
O3	0.0334 (9)	0.0306 (9)	0.0232 (8)	0.0000 (7)	0.0118 (7)	0.0019 (7)
O4	0.0383 (10)	0.0439 (11)	0.0231 (8)	−0.0078 (8)	0.0124 (7)	−0.0015 (7)
O5	0.0724 (14)	0.0362 (10)	0.0262 (9)	−0.0092 (10)	0.0121 (9)	−0.0138 (8)
O6	0.0376 (10)	0.0214 (8)	0.0315 (9)	−0.0086 (7)	0.0036 (7)	−0.0039 (7)
N1	0.0232 (10)	0.0171 (9)	0.0219 (9)	−0.0036 (7)	0.0039 (8)	0.0027 (7)
N2	0.0260 (10)	0.0176 (9)	0.0226 (9)	−0.0041 (8)	0.0069 (8)	0.0029 (7)
N3	0.0166 (9)	0.0137 (8)	0.0181 (9)	−0.0016 (7)	0.0019 (7)	0.0022 (7)
N4	0.0177 (9)	0.0148 (9)	0.0194 (9)	−0.0006 (7)	0.0010 (7)	0.0013 (7)
C1	0.0142 (10)	0.0191 (10)	0.0197 (10)	0.0005 (8)	0.0003 (8)	0.0049 (8)
C2	0.0172 (11)	0.0219 (11)	0.0203 (11)	−0.0007 (9)	0.0046 (9)	0.0018 (9)
C3	0.0175 (11)	0.0172 (10)	0.0270 (11)	−0.0017 (8)	0.0006 (9)	0.0044 (9)
C4	0.0152 (10)	0.0232 (11)	0.0256 (11)	−0.0002 (8)	0.0031 (9)	0.0086 (9)
C5	0.0187 (11)	0.0252 (12)	0.0181 (10)	0.0043 (9)	0.0023 (9)	0.0049 (9)
C6	0.0206 (11)	0.0181 (10)	0.0193 (11)	0.0014 (9)	−0.0008 (9)	0.0025 (8)
C7	0.0317 (13)	0.0234 (12)	0.0373 (14)	−0.0052 (10)	0.0068 (11)	−0.0041 (10)
C8	0.0365 (14)	0.0379 (14)	0.0351 (14)	−0.0037 (11)	0.0067 (11)	0.0187 (11)
C9	0.0519 (17)	0.0324 (14)	0.0218 (12)	0.0057 (12)	0.0101 (11)	−0.0018 (10)
C10	0.0184 (11)	0.0192 (10)	0.0177 (10)	0.0007 (8)	0.0004 (8)	0.0018 (8)
C11	0.0174 (10)	0.0161 (10)	0.0205 (10)	−0.0034 (8)	0.0005 (8)	0.0060 (8)
C12	0.0159 (10)	0.0175 (10)	0.0195 (10)	0.0008 (8)	−0.0010 (8)	0.0049 (8)
C13	0.0155 (10)	0.0203 (11)	0.0181 (10)	0.0007 (8)	−0.0017 (8)	0.0028 (8)
C14	0.0189 (11)	0.0260 (11)	0.0207 (11)	−0.0031 (9)	0.0001 (9)	0.0034 (9)
C15	0.0228 (12)	0.0371 (13)	0.0179 (11)	−0.0019 (10)	0.0032 (9)	0.0006 (9)
C16	0.0325 (13)	0.0310 (13)	0.0204 (11)	−0.0034 (10)	0.0021 (10)	−0.0072 (10)
C17	0.0249 (12)	0.0190 (11)	0.0253 (12)	−0.0028 (9)	−0.0031 (9)	−0.0020 (9)
C18	0.0181 (11)	0.0227 (11)	0.0198 (10)	0.0006 (9)	0.0003 (9)	0.0025 (9)
C19	0.0391 (10)	0.0433 (10)	0.0349 (9)	−0.0054 (8)	0.0068 (8)	0.0050 (8)
C20	0.0504 (11)	0.0500 (11)	0.0512 (11)	0.0040 (9)	0.0021 (9)	−0.0090 (9)
C21	0.0274 (13)	0.0211 (11)	0.0357 (13)	−0.0031 (10)	−0.0010 (10)	0.0035 (10)

### Geometric parameters (Å, °)

**Table:**

S1—C11	1.729 (2)	C6—H6	0.9300
S1—C12	1.766 (2)	C7—H7A	0.9600
O1—C3	1.372 (3)	C7—H7B	0.9600
O1—C7	1.426 (3)	C7—H7C	0.9600
O2—C4	1.374 (3)	C8—H8A	0.9600
O2—C8	1.422 (3)	C8—H8B	0.9600
O3—C5	1.369 (3)	C8—H8C	0.9600
O3—C9	1.427 (3)	C9—H9A	0.9600
O4—C15	1.369 (3)	C9—H9B	0.9600
O4—C19	1.420 (3)	C9—H9C	0.9600
O5—C16	1.372 (3)	C12—C13	1.465 (3)
O5—C20	1.456 (4)	C13—C18	1.394 (3)
O6—C17	1.371 (3)	C13—C14	1.395 (3)
O6—C21	1.426 (3)	C14—C15	1.384 (3)
N1—C10	1.319 (3)	C14—H14	0.9300
N1—N2	1.394 (3)	C15—C16	1.394 (3)
N2—C11	1.313 (3)	C16—C17	1.395 (3)
N3—C11	1.363 (3)	C17—C18	1.382 (3)
N3—N4	1.374 (2)	C18—H18	0.9300
N3—C10	1.379 (3)	C19—H19A	0.9600
N4—C12	1.299 (3)	C19—H19B	0.9600
C1—C2	1.389 (3)	C19—H19C	0.9600
C1—C6	1.400 (3)	C20—H20A	0.9600
C1—C10	1.460 (3)	C20—H20B	0.9600
C2—C3	1.387 (3)	C20—H20C	0.9600
C2—H2	0.9300	C21—H21A	0.9600
C3—C4	1.393 (3)	C21—H21B	0.9600
C4—C5	1.403 (3)	C21—H21C	0.9600
C5—C6	1.380 (3)		
			
C11—S1—C12	87.61 (10)	H9A—C9—H9C	109.5
C3—O1—C7	116.73 (17)	H9B—C9—H9C	109.5
C4—O2—C8	114.35 (17)	N1—C10—N3	107.57 (18)
C5—O3—C9	116.88 (18)	N1—C10—C1	127.0 (2)
C15—O4—C19	116.8 (2)	N3—C10—C1	125.41 (19)
C16—O5—C20	115.3 (2)	N2—C11—N3	110.97 (18)
C17—O6—C21	116.75 (17)	N2—C11—S1	139.70 (16)
C10—N1—N2	109.70 (17)	N3—C11—S1	109.32 (15)
C11—N2—N1	105.61 (16)	N4—C12—C13	121.29 (19)
C11—N3—N4	118.40 (17)	N4—C12—S1	116.85 (16)
C11—N3—C10	106.15 (17)	C13—C12—S1	121.85 (16)
N4—N3—C10	135.43 (17)	C18—C13—C14	120.9 (2)
C12—N4—N3	107.81 (17)	C18—C13—C12	118.23 (19)
C2—C1—C6	120.74 (19)	C14—C13—C12	120.9 (2)
C2—C1—C10	121.14 (19)	C15—C14—C13	119.4 (2)
C6—C1—C10	118.12 (19)	C15—C14—H14	120.3
C3—C2—C1	119.3 (2)	C13—C14—H14	120.3
C3—C2—H2	120.4	O4—C15—C14	124.2 (2)
C1—C2—H2	120.4	O4—C15—C16	115.5 (2)
O1—C3—C2	123.7 (2)	C14—C15—C16	120.3 (2)
O1—C3—C4	115.37 (19)	O5—C16—C15	119.0 (2)
C2—C3—C4	120.9 (2)	O5—C16—C17	121.3 (2)
O2—C4—C3	120.4 (2)	C15—C16—C17	119.6 (2)
O2—C4—C5	120.5 (2)	O6—C17—C18	124.0 (2)
C3—C4—C5	118.98 (19)	O6—C17—C16	115.1 (2)
O3—C5—C6	124.52 (19)	C18—C17—C16	120.8 (2)
O3—C5—C4	114.81 (19)	C17—C18—C13	119.0 (2)
C6—C5—C4	120.67 (19)	C17—C18—H18	120.5
C5—C6—C1	119.37 (19)	C13—C18—H18	120.5
C5—C6—H6	120.3	O4—C19—H19A	109.5
C1—C6—H6	120.3	O4—C19—H19B	109.5
O1—C7—H7A	109.5	H19A—C19—H19B	109.5
O1—C7—H7B	109.5	O4—C19—H19C	109.5
H7A—C7—H7B	109.5	H19A—C19—H19C	109.5
O1—C7—H7C	109.5	H19B—C19—H19C	109.5
H7A—C7—H7C	109.5	O5—C20—H20A	109.5
H7B—C7—H7C	109.5	O5—C20—H20B	109.5
O2—C8—H8A	109.5	H20A—C20—H20B	109.5
O2—C8—H8B	109.5	O5—C20—H20C	109.5
H8A—C8—H8B	109.5	H20A—C20—H20C	109.5
O2—C8—H8C	109.5	H20B—C20—H20C	109.5
H8A—C8—H8C	109.5	O6—C21—H21A	109.5
H8B—C8—H8C	109.5	O6—C21—H21B	109.5
O3—C9—H9A	109.5	H21A—C21—H21B	109.5
O3—C9—H9B	109.5	O6—C21—H21C	109.5
H9A—C9—H9B	109.5	H21A—C21—H21C	109.5
O3—C9—H9C	109.5	H21B—C21—H21C	109.5
			
C10—N1—N2—C11	0.1 (2)	N4—N3—C11—N2	177.96 (17)
C11—N3—N4—C12	0.1 (2)	C10—N3—C11—N2	−0.6 (2)
C10—N3—N4—C12	178.2 (2)	N4—N3—C11—S1	−0.9 (2)
C6—C1—C2—C3	0.8 (3)	C10—N3—C11—S1	−179.49 (13)
C10—C1—C2—C3	−178.50 (19)	C12—S1—C11—N2	−177.3 (3)
C7—O1—C3—C2	10.3 (3)	C12—S1—C11—N3	1.03 (15)
C7—O1—C3—C4	−171.13 (19)	N3—N4—C12—C13	−179.48 (17)
C1—C2—C3—O1	178.88 (19)	N3—N4—C12—S1	0.8 (2)
C1—C2—C3—C4	0.4 (3)	C11—S1—C12—N4	−1.12 (17)
C8—O2—C4—C3	86.6 (3)	C11—S1—C12—C13	179.16 (18)
C8—O2—C4—C5	−97.3 (2)	N4—C12—C13—C18	−2.4 (3)
O1—C3—C4—O2	−4.2 (3)	S1—C12—C13—C18	177.34 (15)
C2—C3—C4—O2	174.44 (19)	N4—C12—C13—C14	176.02 (19)
O1—C3—C4—C5	179.64 (19)	S1—C12—C13—C14	−4.3 (3)
C2—C3—C4—C5	−1.7 (3)	C18—C13—C14—C15	−1.1 (3)
C9—O3—C5—C6	3.6 (3)	C12—C13—C14—C15	−179.42 (19)
C9—O3—C5—C4	−175.7 (2)	C19—O4—C15—C14	−8.6 (3)
O2—C4—C5—O3	5.2 (3)	C19—O4—C15—C16	171.6 (2)
C3—C4—C5—O3	−178.67 (18)	C13—C14—C15—O4	179.6 (2)
O2—C4—C5—C6	−174.19 (18)	C13—C14—C15—C16	−0.6 (3)
C3—C4—C5—C6	2.0 (3)	C20—O5—C16—C15	119.7 (3)
O3—C5—C6—C1	179.86 (19)	C20—O5—C16—C17	−63.8 (3)
C4—C5—C6—C1	−0.9 (3)	O4—C15—C16—O5	−1.3 (3)
C2—C1—C6—C5	−0.5 (3)	C14—C15—C16—O5	178.9 (2)
C10—C1—C6—C5	178.77 (18)	O4—C15—C16—C17	−177.9 (2)
N2—N1—C10—N3	−0.4 (2)	C14—C15—C16—C17	2.3 (4)
N2—N1—C10—C1	−179.76 (19)	C21—O6—C17—C18	−1.0 (3)
C11—N3—C10—N1	0.6 (2)	C21—O6—C17—C16	−179.9 (2)
N4—N3—C10—N1	−177.6 (2)	O5—C16—C17—O6	0.1 (3)
C11—N3—C10—C1	179.98 (19)	C15—C16—C17—O6	176.6 (2)
N4—N3—C10—C1	1.7 (4)	O5—C16—C17—C18	−178.8 (2)
C2—C1—C10—N1	176.3 (2)	C15—C16—C17—C18	−2.3 (4)
C6—C1—C10—N1	−3.1 (3)	O6—C17—C18—C13	−178.1 (2)
C2—C1—C10—N3	−2.9 (3)	C16—C17—C18—C13	0.7 (3)
C6—C1—C10—N3	177.75 (18)	C14—C13—C18—C17	1.1 (3)
N1—N2—C11—N3	0.4 (2)	C12—C13—C18—C17	179.43 (19)
N1—N2—C11—S1	178.7 (2)		

### Hydrogen-bond geometry (Å, °)

**Table:**

*D*—H···*A*	*D*—H	H···*A*	*D*···*A*	*D*—H···*A*
C2—H2···N4	0.93	2.36	3.047 (3)	130
C14—H14···S1	0.93	2.72	3.130 (3)	108
C19—H19B···O3^i^	0.96	2.53	3.384 (2)	148
C21—H21B···O1^ii^	0.96	2.43	3.331 (3)	156
C19—H19C···Cg3^iii^	0.96	3.30	4.057 (3)	137

Symmetry codes: (i) *x*+1, *y*, *z*−1; (ii) −*x*, −*y*, −*z*+2; (iii) −*x*+1, −*y*+1, −*z*.
